# OA05.02. Relationships among well-being and wellness behaviors over time in residents in eight family medicine residencies

**DOI:** 10.1186/1472-6882-12-S1-O18

**Published:** 2012-06-12

**Authors:** S Dodds, A Brooks, J Woytowicz, P Cook, R Benn, V Maizes, P Lebensohn

**Affiliations:** 1 University of Arizona Center for Integrative Medicine, Tucson, USA; 2 Maine-Dartmouth, Augusta, USA; 3 University of Michigan Health Center, Ann Arbor, USA

## Purpose

To present findings on relationships among dimensions of well-being and wellness behaviors in family medicine residents participating in the Integrative Medicine in Residency (IMR) program through the last two years of training.

## Methods

Residents in the 2011 graduating class of the IMR (n=56) were assessed at the beginning of PGY2 and PGY3 and at graduation. Measures were self-administered online and included established measures of well-being: perceived stress, burnout (emotional exhaustion, depersonalization), depression, and satisfaction with life. Wellness behaviors assessed included restful sleep, nutrition, physical activity, mind-body activities, being in nurturing relationships, being outdoors in nature, and alcohol use. Stepwise regression analysis examined relationships between wellness behaviors and each well-being measure at each timepoint.

## Results

In both PGY2s (n=52) and PGY3s (n=38), restful sleep was associated with less perceived stress (p=0.003; p=0.01), greater life satisfaction (p=0.007; p=0.007), less depression (p=0.002; p=0.041), and less emotional exhaustion (p=0.001; p<0.001). In PGY2s, more time in nurturing relationships was associated with greater life satisfaction (p=0.039). In PGY3s, more frequent exercise was associated with less depression (p=0.003) and greater life satisfaction (p=0.014). By graduation (n=42), sleep was associated with less emotional exhaustion (p=0.006). Spending more time outdoors in nature was associated with lower perceived stress (p=0.002), less depression (p=0.026), and lower depersonalization (p=0.003). Nurturing relationships were associated with greater life satisfaction (p<0.001). More alcohol use was associated with less perceived stress (p=0.001). Using a variety of mind-body wellness behaviors was associated with greater depression in PGY2s (p=0.015), and with emotional exhaustion at graduation (p=0.045).

## Conclusion

Sleep, nurturing relationships, exercise, and time outdoors in nature were most frequently associated with well-being among Family Medicine residents. This study is the first to describe these relationships for residents who participated in the IMR, and points to the importance of addressing well-being and wellness behaviors during the formative time of graduate medical education.

